# Redox index of Cys-thiol residues of serum apolipoprotein E and its diagnostic potential

**DOI:** 10.1042/BSR20211060

**Published:** 2021-08-05

**Authors:** Kazuyoshi Yamauchi, Chiaki Taira, Yasushi Kawakami

**Affiliations:** 1 Department of Laboratory Medicine, Faculty of Medicine, University of Tsukuba, Tsukuba, Japan; 2 Department of Biomedical Laboratory Sciences, Shinshu University School of Medicine, Matsumoto, Japan

**Keywords:** apolipoprotein E, atherosclerosis, band shift assay, remnant lipoprotein, reversible oxidation, type 2 diabetes

## Abstract

Background: The redox modulation of Cys-thiol participates in various pathophysiological processes. We explored the proper index for estimating the redox status of Cys-thiol of serum apolipoprotein E (apoE), named “redox-IDX-apoE,” which is necessary to understand the redox biology of age-related diseases.

Methods: The fractions of the reduced form (red-), reversible oxidized form (roxi-), and irreversibly oxidized form (oxi-) apoE in serum, obtained from the patients with no apparent disease (controls, *n*=192) and with atherosclerosis and type 2 diabetes (patients, *n*=16), were measured by a band-shift assay using a maleimide compound. Redox-IDX-apoE candidates were determined by calculating the values of these fractions and the total apoE concentration.

Results: Cys number of apoE significantly increased for the ratio of roxi-apoE to total-apoE (roxi/total) (E2/E3>E3/E3>E3/E4) but decreased for the ratios of red-apoE to roxi-apoE (red/roxi) and [red-apoE + oxi-apoE] to roxi-apoE ([red + oxi]/roxi) (E2/E3<E3/E3<E3/E4). Considering the subjects with apoE3/E3, these ratios were independent of age and sex. Roxi/total showed negative correlations with serum triglyceride (TG) and HbA1c levels, while both red/roxi and [red + oxi]/roxi showed significant positive correlations with them. However, red/roxi and [red + oxi]/roxi in patients were significantly lower than those in controls, although serum TG and HbA1c levels in the patients were significantly higher than those in controls.

Conclusion: The redox status of serum apoE-Cys-thiol is closely involved in the metabolism of TG-rich lipoproteins and glucose. The appropriate use of redox-IDX-apoE could be helpful in the diagnosis and prognosis of age-related diseases and in understanding the underlying mechanisms.

## Introduction

The establishment of prevention, early diagnosis, and treatment of age-related diseases is an urgent issue in a rapidly aging society. It is well known that age-related diseases, such as atherosclerotic diseases and Alzheimer’s disease (AD), are caused by oxidative stress [1]. Additionally, apolipoprotein E (apoE) has long been regarded as a key molecule contributing to the pathogenesis of several diseases [2]. Therefore, numerous studies focusing on these two points have been performed to clarify the pathophysiological mechanisms specific to age-related diseases; however, the details remain obscure.

Apolipoprotein (apo) E participates in cholesterol transport and metabolism as the main constituent of plasma lipoproteins and a ligand for the low-density lipoprotein (LDL) receptor [3]. ApoE has three major isoforms (E2, Cys112/Cys158; E3, Cys112/Arg158; E4, Arg112/Arg158), which are derived from Cys-Arg interchanges at residues 112 and/or 158 [3]. These interchanges provide a structural and functional basis for these three isoforms [4] and make a noticeable difference in the existing form of plasma apoE among these three isoforms. Namely, apoE2 and apoE3 exist in the plasma not only in monomeric forms but also in disulfide-linked complexes, such as homodimer, apoE-AII complex, and apoAII-E2-AII complex, whereas apoE4 exists only in the monomeric form [5,6].

These properties, based on Cys-Arg interchanges, have been considered to contribute to the development of various age-related diseases [1,4,7]. Specifically, apoE4, an isoform without a Cys residue, is a risk factor for various atherosclerotic diseases, such as cardiovascular disease and cerebral infarction [2,8] and sporadic late-onset AD [4,7]. In contrast, apoE2, an isoform with two Cys residues, is a causative factor of type III hyperlipidemia since apoE2 is defective in LDL receptor binding affinity owing to the effect of Cys158 [9].

Cys is one of the least abundant amino acids. The thiol group of Cys in proteins imparts functional sites with specialized properties, such as nucleophilicity, high-affinity metal binding, and disulfide bond formation [10]. Therefore, Cys-thiol, as one of the main targets of post-translational redox-mediated modifications, has roles in various physiological functions (e.g. as regulatory switches in signal pathways, modulation of transcription and protein expression, maturation of proteins, and protection from oxidative stress) [11–13].

Redox-mediated modifications of Cys-thiols are classified into reversible and irreversible oxidations. The irreversible oxidation of Cys-thiol is predicted to cause loss of function, leading to degradation of the modified protein [14,15]. Conversely, the temporary alteration of a protein due to reversible oxidation protects the protein from irreversible detrimental changes and modulates protein function [16]. Consequently, redox-mediated modifications of Cys-thiol participate in the pathogenesis of various diseases [17]. Based on these evidences, there is no doubt that the redox status of the apoE (apoE-Cys-thiol) Cys-thiol also affects its pathophysiological functions. We believe that some redox modulations of Cys-thiol confer detrimental or beneficial properties to apoE2 or apoE3, which affects the development of various apoE-related diseases, especially in apoE4 non-carrier patients.

In our previous study, we demonstrated that the apoE3-AII complex is beneficial for maintaining the apoE3 redox status by preventing changes to the irreversibly oxidized form [18]. We also recently reported that the interactions of apoE2 and apoE3, especially apoE2, with lipids are markedly enhanced by the formation of apoE-AII and apoAII-E2-AII complexes [19]. However, in addition to our studies, few studies focusing on the redox status of apoE-Cys-thiol have been conducted; thus, its precise physiological meaning is still obscure. Understanding whether or how the redox status of apoE-Cys-thiol is related to the development of age-related diseases is necessary to establish an adequate method for its estimation.

In the present study, we explored the proper index for estimating the redox status of apoE-Cys-thiol, named redox-IDX-apoE, and assessed its clinical availability by investigating its pathophysiological variance.

## Materials and methods

### Materials

Horseradish peroxidase (HRP)-conjugated goat anti-apoE polyclonal antibody was supplied by the Academy Bio-medical Company, Inc. (Houston, TX, U.S.A.). Photocleavable maleimide-conjugated polyethylene glycol (PEG-PC-Mal) was purchased from Dojindo Molecular Technologies, Inc. (Kumamoto, Japan). All other chemicals used were of the highest grade.

### Subjects

Two hundred and eighty serum samples were obtained from outpatients, who underwent a health screening in Tsukuba University Hospital, and were verified to be healthy (without any disease) (*n*=192, 52.7 ± 17.1 years) or with atherosclerosis combined with type 2 diabetes (*n*=16, 56.8 ± 19.6 years). All these patients showed normal results on the following serum laboratory tests: creatinine (male, 0.65–1.07 mg/dL; female, 0.46–0.79 mg/dL), uric acid (male, 3.7–7.8 mg/dL; female, 2.6–5.5 mg/dL), AST (13–30 U/L), ALT (male, 10–42 U/L; female, 7–23 U/L), LD (124–222 U/L), and γGT (male, 13–64 U/L; female, 9–32 U/L). All serum samples were aliquoted in small volumes and then stored at -80°C until analysis, in order to avoid artificial oxidation caused by repeated freeze–thaw cycles based on the results of our preliminary experiment. The present study was conducted in accordance with the principles of the Declaration of Helsinki and was approved by the Tsukuba University Ethics Committee (H28-075). Written informed consent was obtained from all patients.

### ApoE phenotyping

The serum apoE phenotype was determined by isoelectric focusing and immunoblot analysis, as described previously [20].

### Determination of candidates of redox-IDX-apoE

**Figure 1:**
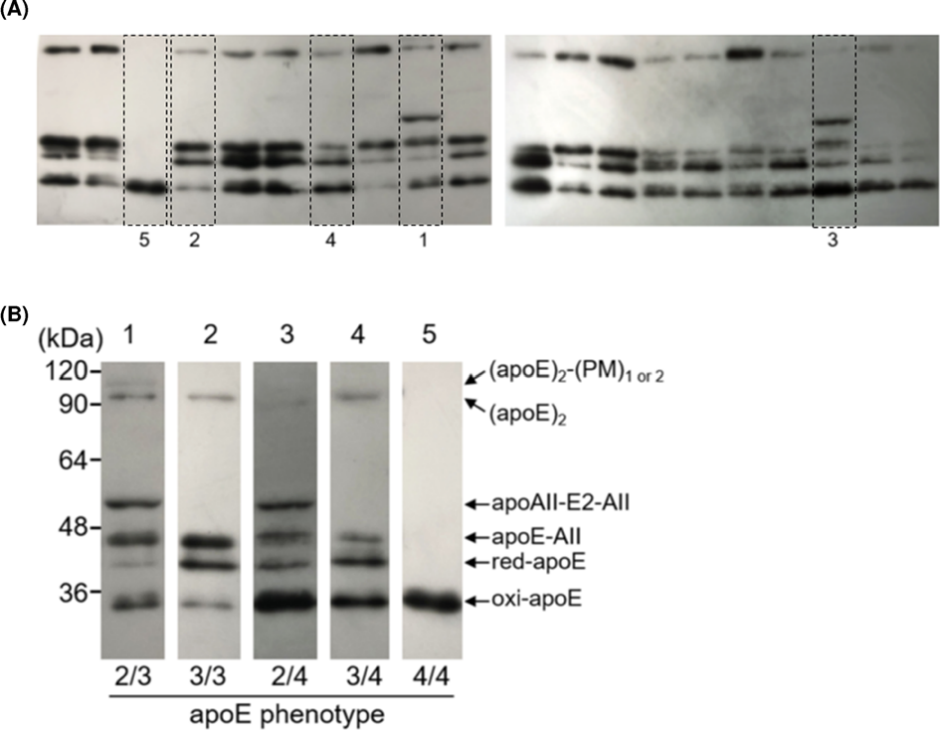
Typical pattern of band-shift assay for sera with different apoE phenotypes using PEG-PC-Mal (PM) Each serum sample, diluted to approximately 0.05 mg/dL, was incubated with 1.0 mmol/L PM for 30 min at 37°C. The reaction was later quenched by the addition of non-reducing Laemmli buffer, and SDS-PAGE was performed using a 10% gel. After electrophoresis, the gel was UV-irradiated for 15 min, after which the separated proteins were detected by immunoblot analysis using a conjugated goat anti-apoE polyclonal antibody [21]. Typical patterns were picked up from the original blot patterns (**A**) and were rearranged (**B**). Lane 1, apoE2/E3 (serum apoE concentration, 2.3 mg/dL); Lane 2, apoE3/E3 (2.1 mg/dL); Lane 3, apoE2/4 (2.9 mg/dL); Lane 4, apoE3/E4 (2.9 mg/dL); Lane 5, apoE4/E4 (1.4 mg/dL).

The redox status of apoE was analyzed with a band-shift assay using PEG-PC-Mal (PM), according to our previous study [19,21]. The specific bands, probed with HRP-conjugated anti-apoE polyclonal antibody, were developed using an ECL detection kit (Nacalai Tesque, Inc., Kyoto, Japan) and analyzed using ImageJ 1.45 software from the National Institutes of Health. At least two independent band-shift assays were performed. The typical pattern of the present assay is shown in Figure 1. As described previously [19,21], the 40-kDa band (apoE-(PM)_1_) was defined as the reduced form of apoE (red-apoE), while the monomeric form (35-kDa unlabeled apoE) was termed as the irreversibly oxidized form of apoE (oxi-apoE). The concentrations of red- and oxi-apoE were determined by multiplying the fraction ratios by the total apoE concentration. In the present method, irreversibly oxidized apoE2 and apoE3 are indistinguishable from apoE4 because apoE4 is also detected at the position of monomeric apoE. Thus, we also determined the concentration of the reversible oxidized form of apoE (roxi-apoE) (i.e. the total concentration of homodimers, heterodimers, and PM conjugates of these disulfide-linked complexes) by subtracting the oxi-apoE concentration from the total apoE concentration. In addition, the following redox ratios of apoE were calculated from the concentrations of total-, red-, roxi-, and oxi-apoE: ratios of red-apoE to total apoE (red/total), roxi-apoE to total apoE (roxi/total), oxi-apoE to total apoE (oxi/total), red-apoE to oxi-apoE (red/oxi), red-apoE to roxi-apoE (red/roxi), roxi-apoE to oxi-apoE (roxi/oxi), red-apoE to [roxi-apoE + oxi-apoE] (red/[roxi + oxi]), [red-apoE + roxi-apoE] to oxi-apoE ([red+roxi]/oxi), and [red-apoE + oxi-apoE] to roxi-apoE ([red+oxi]/roxi).

### Other assays

The concentrations of serum apolipoproteins (apoAI, apoAII, and apoE), lipids (total cholesterol [TC], high-density lipoprotein cholesterol [HDL-C], LDL-C, and TG), and CRP were determined by turbidimetric immunoassay, enzymatic assay, and latex turbidimetric immunoassay, respectively. HbA1c values were measured by cation-exchange high-performance liquid chromatography. The concentration of non-HDL-C was calculated as TC minus HDL-C.

### Statistical methods

Statistical analysis was performed using BellCurve for Excel (Social Survey Research Information Co., Ltd. Tokyo, Japan). All laboratory parameters and ages of subjects are presented as the mean ± standard error (SE) and mean ± standard deviation (SD), respectively. One-way ANOVA was used to compare the differences in each laboratory test result among apoE phenotype groups and the differences in each redox ratio among apoE phenotype groups or between male and female subject groups. Univariate and multivariate regression analyses were performed to assess the relationship between each redox-IDX-apoE and each laboratory test result, and to determine the independent factors that affect each redox-IDX-apoE. Statistical significance was set at *P<*0.05.

## Results

### Effect of apoE phenotype on the candidates of redox-IDX-apoE

**Figure 2:**
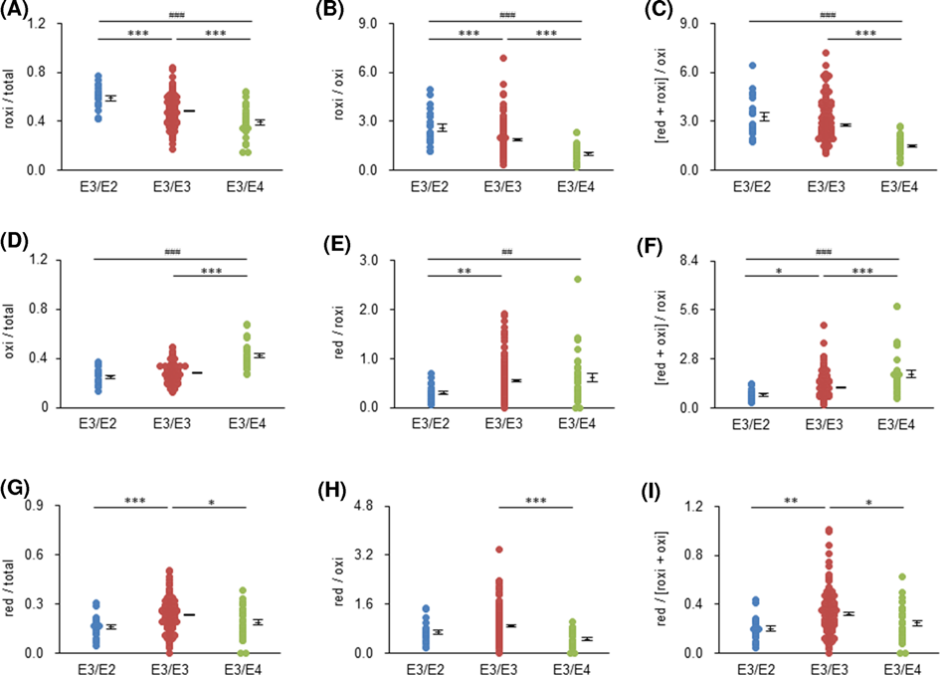
Comparisons of the candidates of redox-IDX-apoE among apoE phenotype groups The differences in roxi/total (**A**), roxi/oxi (**B**), [red + roxi]/oxi (**C**), oxi/total (**D**), red/roxi (**E**), [red + oxi]/roxi (**F**), red/total (**G**), red/oxi (**H**), and red/[roxi + oxi] (**I**) among the apoE phenotype groups were compared using one-way ANOVA. Data are expressed as the mean ± SE. *, *P*<0.05; **, *P*<0.01; ***, *P*<0.001, compared with the group of subjects with apoE3/E3. ^#^, *P*<0.05; ^##^, *P*<0.01; ^###^, *P*<0.001, compared with the group of subjects with E2/E3. ^†^, *P*<0.05, compared with the apoE3/E4 group.

We evaluated the effect of the apoE phenotype, namely the number of Cys residues per two apoE molecules, on various redox-IDX-apoE candidates (Table 1). The roxi-apoE concentrations in serum with apoE2/E3 were significantly higher than those with apoE3/E3 or apoE3/E4 (*P*<0.001), while the oxi-apoE concentrations in serum with apoE3/E4 were significantly lower than those in serum with apoE3/E3 (*P*<0.001). However, it was not certain whether these differences were due to only the number of Cys residues in apoE, since total-apoE concentration itself, although not statistically significant, was different among phenotype groups. Hence, we narrowed down the candidates of redox-IDX-apoE to the redox ratios of apoE, calculated from concentrations of total-, red-, roxi-, and oxi-apoE, and assessed the effect of the number of Cys residues in apoE on these ratios. Serum results with apoE2/E4 were excluded from this evaluation because the sample size was small. Roxi/total, roxi/oxi, and [red+roxi]/oxi were clearly proportional to the number of Cys residues in apoE and varied in the order E2/E3>E3/E3>E3/E4 (Figure 2A–C). Roxi/total, roxi/oxi, and [red+roxi]/oxi in the serum with apoE2/E3 were approximately 1.5-fold (*P*<0.001), 2.5-fold (*P*<0.001), and 2.2-fold (*P*<0.001) higher than those in the serum with apoE3/E4, respectively. In contrast, oxi/total, red/roxi, and [red+oxi]/roxi decreased with increasing Cys number of apoE and varied in the rank order E2/E3<E3/E3< E3/E4 (Figure 2D–F). Oxi/total, red/roxi, and [red+oxi]/roxi in the serum with apoE3/E4 were approximately 1.7-fold (*P*<0.001), 2.1-fold (*P*<0.01), and 2.6-fold (*P*<0.001) higher than those in the serum with apoE2/E3, respectively. Unlike the six ratios, red/total, red/oxi, and red/[roxi + oxi] were not affected by the number of Cys residues in apoE. These three ratios in the serum with apoE3/E3 were significantly higher than those in the serum with other phenotypes (Figure 2G–I). Hence, to avoid the effect of the number of Cys residues in apoE, we conducted the following analysis with a focus on subjects with apoE3/E3.

**Table 1:** Comparison of the candidates of redox-IDX-apoE among apoE phenotype groups

apoE phenotype	E2/E3	E3/E3	E2/E4	E3/E4	E4/E4
Cys number/apoE molecules	1.5	1	1	0.5	0
*n* (male/female)	21 (9/12)	218(92/126)	4 (1/4)	34 (22/12)	3 (2/1)
Age	58.3 ± 17.5	51.8 ± 17.0	53.8 ± 18.5	56.0 ± 16.7	48.7 ± 15.0
apoAI (mg/dL)	147.6 ± 6.3	146.6 ± 2.1	129.3 ± 8.7	149.4 ± 5.1	141.3 ± 6.0
apoAII (mg/dL)	31.6 ± 1.9	31.7 ± 0.5	30.6 ± 2.1	32.7 ± 1.4	35.6 ± 3.4
HDL-C (mg/dL)	61.2 ± 4.6	56.8 ± 1.3	48.2 ± 2.7	56.0 ± 3.0	49.2 ± 8.3
LDL-C (mg/dL)	85.4 ± 6.0 ^*^	106.0 ± 2.1	136.8 ± 18.2 ^##^	98.7 ± 4.2	96.0 ± 9.3
Non-HDL-C (mg/dL)	115.6 ± 7.9	136.7 ± 2.6	172.1 ± 24.0 ^#^	135.1 ± 7.6	131.5 ± 10.9
TG (mg/dL)	147.4 ± 30.3	154.9 ± 7.3	186.5 ± 56.2	184.3 29.9	153.3 ± 38.8
CRP (mg/dL)	0.288 ± 0.112	0.342 ± 0.069	0.312 ± 0.099	0.460 ± 0.105	0.223 ± 0.199
HbA1c (%)	6.00 ± 0.22	5.92 ± 0.08	5.53 ± 0.16	5.80 ± 0.17	5.57 ± 0.22
Total-apoE (mg/dL)	3.63 ± 0.33	2.91 ± 0.09	3.55 ± 0.18	2.89 ± 0.25	1.37 ± 0.09
Red-apoE (mg/dL)	0.56 ± 0.07	0.69 ± 0.03	0.52 ± 0.06	0.58 ± 0.08	
roxi-apoE (mg/dL)	2.16 ± 0.21 ^***^	1.40 ± 0.05	1.76 ± 0.11	1.11 ± 0.11 ^###^	N/A
oxi-apoE (mg/dL)	0.91 ± 0.11	0.82 ± 0.03	1.26 ± 0.09	1.20 ± 0.11 ^***^	
red/total	0.161 ± 0.015 ^***^	0.233 ± 0.006	0.147 ± 0.014	0.187 ± 0.016 ^*^	
roxi/total	0.589 ± 0.022 ^***^	0.485 ± 0.007	0.497 ± 0.024	0.392 ± 0.023 ^***###^	
oxi/total	0.249 ± 0.014	0.282 ± 0.004	0.356 ± 0.015 ^#^	0.421 ± 0.017 ^***###^	
red/oxi	0.685 ± 0.075	0.883 ± 0.031	0.412 ± 0.035	0.463 ± 0.042 ^***^	
red/roxi	0.297 ± 0.037 ^**^	0.544 ± 0.022	0.301 ± 0.041	0.613 ± 0.087 ^##^	N/A
roxi/oxi	2.614 ± 0.237 ^***^	1.884 ± 0.057	1.411 ± 0.122 ^#^	1.024 ± 0.088 ^***###^	
red/[roxi + oxi]	0.200 ± 0.022 ^**^	0.322 ± 0.011	0.173 ± 0.019	0.246 ± 0.025 ^*^	
[red + roxi]/oxi	3.298 ± 0.265	2.767 ± 0.068	1.824 ± 0.117 ^#^	1.487 ± 0.090 ^***###^	
[red + oxi]/roxi	0.747 ± 0.070^*^	1.179 ± 0.040	1.026 ± 0.100 ^†^	1.941 ± 0.225 ^***###^	

All parameters and ages of subjects are presented as the mean ± SE and the mean ± SD, respectively.

red, reduced form; roxi, reversible oxidized form; oxi, oxidized form. N/A, not applicable.

*, *P*<0.05; **, *P*<0.01; ***, *P*<0.001 (vs E3/E3). ^#^, *P*<0.05; ^##^, *P*<0.01; ^###^, *P*<0.001 (vs E2/E3). ^†^, *P*<0.05 (vs E3/E4).

### Effect of age on the candidates of redox-IDX-apoE

**Figure 3:**
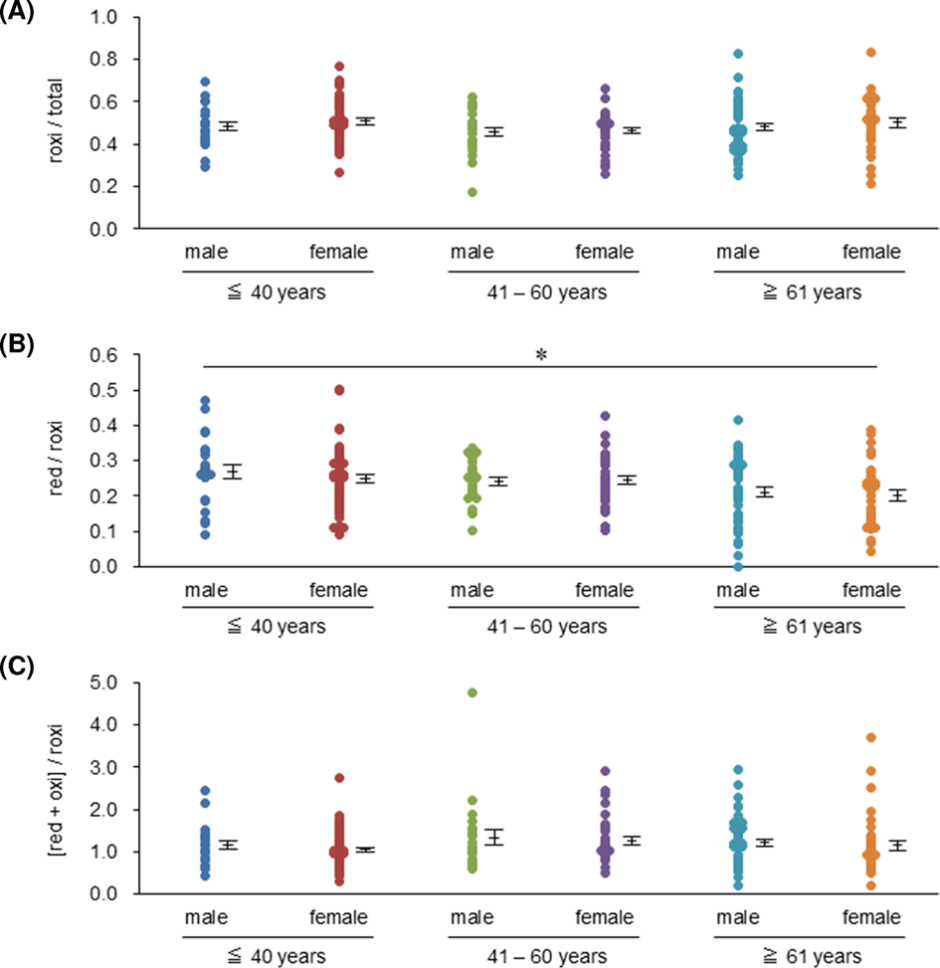
Comparisons of the candidates of redox-IDX-apoE among age-stratified male and female groups The differences in roxi/total (**A**), red/roxi (**B**), and [red + oxi]/roxi (**C**) among the apoE3/E3 male subjects and the apoE3/E3 female subjects, which were divided into three groups by age (≤ 40 years, 41–60 years, ≥ 61 years), were compared using one-way ANOVA. Data are expressed as the mean ± SE; *, *P*<0.05.

We assessed the effect of age on the candidates for redox-IDX-apoE in the serum with apoE3/E3 according to sex (Table 2). Oxi/total levels were significantly increased by aging, regardless of sex (*r* = 0.368, *P*<0.001). In contrast, red/total, red/oxi, and [red + roxi]/oxi levels significantly decreased with increasing age, regardless of sex (*r* = -0.246, *P*<0.001 for red/total; *r* = -0.358, *P*<0.001 for red/oxi; *r* = -0.302, *P*<0.001 for [red + roxi]/oxi). Red/[roxi + oxi] in males was also significantly decreased with increasing age (*r* = -0.312, *P*<0.005), whereas those in females showed a decrease in inclination, but the difference was not statistically significant (*r* = -0.113). In contrast, no significant correlation was observed between roxi/total, red/roxi, and [red + oxi]/roxi with age. We then compared each of these three ratios among male and female subjects, which were divided into three groups by age (≤40, 41–60 years, and ≥61 years). No significant differences in roxi/total (Figure 3A), red/roxi (except for the slight significant difference between the male subjects aged ≤40 years and female subjects aged ≥61 years (Figure 3B), and [red + oxi]/roxi (Figure 3C) were observed between male and female subjects. Based on these results, to avoid the effects of sex and age as confounding factors, we selected roxi/total, red/roxi, and [red + oxi]/roxi as the redox-IDX-apoE.

**Table 2:** Effect of age and sex on the candidates of redox-IDX-apoE in the serum with apoE3/E3

	Correlation between age and each redox-Idx-apoE					
Candidates of redox-Idx-apoE	Male (*n*=92)		Female (*n*=126)		Male + Female (*n*=218)	
	*r*	*P*	*r*	*P*	*r*	*P*
red/total	-0.265	<0.05	-0.243	0.01	-0.246	<0.001
roxi/total	0.005	NS	-0.018	NS	-0.025	NS
oxi/total	0.328	<0.005	0.372	<0.001	0.368	<0.001
red/oxi	-0.375	<0.001	-0.343	<0.001	-0.358	<0.001
red/roxi	-0.109	NS	-0.090	NS	-0.088	NS
roxi/oxi	-0.142	NS	-0.156	NS	-0.167	<0.05
red/[roxi + oxi]	-0.312	<0.005	-0.113	NS	-0.236	<0.001
[red + roxi]/oxi	-0.289	<0.005	-0.287	<0.005	-0.302	<0.001
[red + oxi]/roxi	0.050	NS	0.065	NS	0.074	NS

NS, not significant.

### Determination of the factors affecting redox-IDX-apoE

We statistically determined the factors that affected redox-IDX-apoE. First, we assessed the relationship between each redox-IDX-apoE and each laboratory test result (apoAI, apoAII, HDL-C, LDL-C, non-HDL-C, TG, TG to HDL-C ratio (TG/HDL-C), CRP, and HbA1c) by simple linear regression analysis (Supplementary Table S1). Roxi/total was positively correlated with HDL-C levels (*r* = 0.236, *P*<0.001), whereas it was negatively correlated with non-HDL-C levels (*r* = -0.275, *P*<0.001), TG levels (*r* = -0.483, *P*<0.001), TG/HDL-C (*r* = -0.384, *P*<0.001), and HbA1c levels (*r* = -0.192, *P*<0.005). Both of red/roxi and [red + oxi]/roxi showed positive correlations with non-HDL-C levels (*r* = 0.247, *P*<0.001 for red/roxi; *r* = 0.237, *P*<0.001 for [red + oxi]/roxi), TG levels (*r* = 0.542, *P*<0.001 for red/roxi; *r* = 0.515, *P*<0.001 for [red + oxi]/roxi), TG/HDL-C (*r* = 0.447, *P*<0.001 for red/roxi; *r* = 0.425, *P*<0.001 for [red + oxi]/roxi), and HbA1c levels (*r* = 0.182, *P*<0.01 for red/roxi; *r* = 0.183, *P*<0.01 for [red + oxi]/roxi), whereas it showed negative correlation with HDL-C levels (*r* = -0.277, *P*<0.001 for red/roxi; *r* = -0.256, *P*<0.001 for [red + oxi]/roxi).

Next, we performed multiple regression analysis with each redox-IDX-apoE as the objective variable and with the factors narrowed down by the above-mentioned linear regression analysis as explanatory variables that affected each redox-IDX-apoE. However, non-HDL-C and TG/HDL-C were excluded from this analysis because they showed a linear combination with HDL-C and TG levels. In this analysis, HbA1c and serum TG levels were independently associated with redox-IDX-apoE (Table 3).

**Table 3:** Univariate and multivariate analysis for the redox-IDX-apoE in the serum with apoE3/E3

Objective variable	Explanatory variable	Univariate analysis					Multivariate analysis				
		*B*	SE	β	*t*	*P*	B	SE	β	*t*	*P*
	HDL-C (mg/dL)	0.0013	0.0004	0.236	3.5693	<0.001	NS				
	non-HDL-C (mg/dL)	-0.0008	0.0002	-0.2749	-4.2028	<0.001	†				
roxi/total	TG (mg/dL)	-0.0005	0.0001	-0.4832	-8.0926	<0.001	-0.0005	0.0001	-0.4649	-7.7552	<0.001
	TG/HDL-C	0.0499	0.0050	0.5619	10.0053	<0.001	†				
	HbA1c (%)	-0.0181	0.0063	-0.1915	-2.8668	<0.005	-0.0116	0.0057	-0.1222	-2.0381	<0.05
	HDL-C (mg/dL)	-0.0047	0.0011	-0.2766	-4.2295	<0.001	NS				
	non-HDL-C (mg/dL)	0.0021	0.0006	0.2470	3.7455	<0.001	†				
red/roxi	TG (mg/dL)	0.0017	0.0002	0.5421	9.4584	<0.001	0.0014	0.0002	0.472	7.4124	<0.001
	TG/HDL-C	0.0824	0.0053	0.7254	15.5221	<0.001	†				
	HbA1c (%)	0.0534	0.0196	0.1825	2.7287	<0.01	0.0292	0.107	0.0944	0.0994	<0.05
	HDL-C (mg/dL)	-0.0077	0.0020	-0.2563	-3.8972	<0.001	NS				
	non-HDL-C (mg/dL)	0.0036	0.001	0.2374	3.5919	<0.001	†				
[red + oxi]/roxi	TG (mg/dL)	0.0028	0.0003	0.5152	8.8141	<0.001	0.0027	0.0003	0.4991	8.4886	<0.001
	TG/HDL-C	0.1089	0.0111	0.7181	15.2018	<0.001	†				
	HbA1c (%)	0.0949	0.0347	0.1829	2.7341	<0.01	0.0560	0.0305	0.1078	1.8329	<0.05

†, Non-HDL-C level and TG/HDL-C were excluded from the objective variable of multivariate analysis, since they showed the linear combination with HDL-C and TG level.

NS, not significant.

### Effect of atherosclerosis on redox-IDX-apoE

To investigate the clinical significance and usefulness of roxi/total, red/roxi and [red + oxi]/roxi as the redox-IDX-apoE, we selected 16 subjects with atherosclerosis combined with type 2 diabetes (56.8 ± 19.6 years) and 38 subjects with normolipidemia and no apparent disease (controls, 54.7 ± 17.0 years) from the above-mentioned 218 subjects with apoE3/E3. We then compared each of these ratios between the subjects with atherosclerosis and controls.

**Figure 4:**
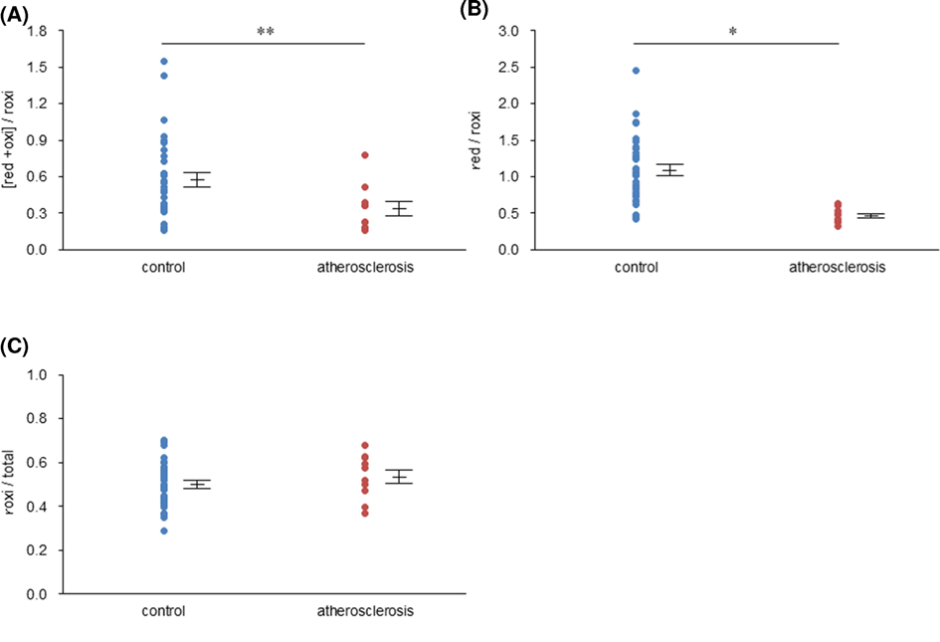
Effect of atherosclerosis on the redox-IDX-apoE The differences in roxi/total (**A**), red/roxi (**B**), and [red + oxi]/roxi (**C**) between the apoE3/E3 subjects with atherosclerosis combined with type 2 diabetes (*n* = 16, 56.8 ± 19.6 years) and the apoE3/E3 subjects with normolipidemia and no apparent disease (*n* = 38, 54.7 ± 17.0 years) were compared using the Mann–Whitney *U* test. Data are expressed as the mean ± SE; *, *P*<0.05; **, *P*<0.001.

The effects of atherosclerosis on roxi/total, red/roxi, and [red + oxi]/roxi conflicted with the results described in above section. Red/roxi and [red + oxi]/roxi were 0.6-fold lower (*P*<0.05) and 0.4-fold lower (*P*<0.001) in subjects with atherosclerosis than in controls, respectively (Figure 4A,B). In contrast, the roxi/total ratio tended to be higher in subjects with atherosclerosis than in controls (Figure 4C), even though serum TG and HbA1c levels in subjects with atherosclerosis were significantly higher than those in controls (*P*<0.005 for TG; *P*<0.01 for HbA1c, Supplementary Table S2).

## Discussion

Redox-IDX-apoE is considered an appropriate index for the pathophysiological assessment of the redox status of serum apoE-Cys-thiol. In the present study, we aimed to determine the use of redox-IDX-apoE as a novel biomarker for age-related diseases, such as atherosclerotic diseases.

The concentrations of red-, roxi-, and oxi-apoE are not appropriate indices because they are directly affected by the total apoE concentration. However, when the redox ratios, calculated from those concentrations, were used as indices, the phenotype-specific effects on the redox status of serum apoE-Cys-thiol could be explicitly estimated. In particular, the subjects with apoE2/E3 showed the highest level of roxi/total and the lowest levels of oxi/total, red/total, red/roxi (which reflects the content ratio of reduced monomeric form to reversible oxidized or reducible polymeric form), and [red + oxi]/roxi (which reflects the content ratio of total monomeric form to reducible polymeric form) among phenotype groups. Therefore, serum apoE2 exists as a reducible polymeric molecule rather than as a reduced monomeric molecule, thereby preventing the change to the irreversibly oxidized monomeric molecule. These properties of the subjects with apoE2/E3 were completely opposite to those of subjects with apoE3/E4. Recently, we reported that the formation of the reversible oxidized form of apoE, such as a homodimer and apoE-AII complex, is beneficial for maintaining the apoE3 redox status by preventing changes to the irreversibly oxidized form [18]. The present results can also be explained by this notion.

Previous studies have shown that the cys-thiol group of a protein is susceptible to aging-associated oxidative stress, which consequently affects the pathophysiological function of the corresponding protein [17,22]. In the analysis focusing on only subjects with apoE3/E3, regardless of sex, oxi/total positively correlated with age, whereas red/total, red/oxi, and [red + roxi]/oxi were negatively correlated with age, indicating that the level of irreversibly oxidized monomeric apoE molecules increased, whereas that of the reduced monomeric molecule decreased with aging. These findings suggest that the redox status of serum apoE-Cys-thiol must also be altered by aging. Unlike the six ratios, roxi/total, [red + oxi]/roxi, and red/roxi were independent of age and sex, the most common confounding factors. Considering these results, we selected these three ratios as the potent redox-IDX-apoE.

We previously reported that although serum red-apoE displays a preference for VLDL, its levels showed a significant correlation with serum HDL-C levels [21]. This finding led us to the notion that the redox status of apoE-Cys-thiol affects both the metabolism of VLDL and HDL, and their linkage. The present data of significant correlations of roxi/total, [red + oxi]/roxi, and red/roxi with serum lipid levels, and TG/HDL-C support the above notion. Each of the three ratios showed a significant correlation with HbA1c levels. Previous studies have demonstrated that HbA1c levels are positively associated with circulating TG levels, reflecting hypertriglyceridemia caused by type 2 diabetes [23]. Based on this finding, the redox status of serum apoE-Cys thiol may also be involved in disorders of glucose metabolism or accompanying dyslipidemia. In addition to hypertriglyceridemia, hypo-HDL-cholesterolemia is a typical and common dyslipidemia induced by insulin resistance in patients with type 2 diabetes [24]. Thus, TG/HDL-C, an atherogenic index [25], is also useful as a surrogate marker for insulin resistance [26,27]. In the present study, roxi/total was negatively correlated with TG/HDL-C, whereas [red +oxi]/roxi and red/roxi showed a positive correlation with TG/HDL-C, indicating that these ratios could also reflect the degree of insulin resistance, that is, the reduction of roxi/total and the increment of [red +oxi]/roxi or red/roxi may be indicative of exacerbation of insulin resistance.

The results of the multivariate analysis indicated that the redox status of serum apoE-Cys-thiol was more strongly associated with the metabolism of TG-rich lipoproteins than HDL. TG-rich lipoproteins, such as chylomicrons and VLDL, are catabolized by lipoprotein lipase (LPL) [24,28]. Subsequently, the remnants are rapidly removed from circulation via receptor-mediated clearance [24,28]. LPL also enhances this process along with apoE, which is a critical ligand for the clearance of remnant lipoproteins [28]. Additionally, the activity of LPL is regulated by insulin [29]. Therefore, the exacerbation of insulin resistance causes stagnation of remnant lipoproteins, followed by the formation of atherosclerotic lesions [24,30]. In contrast, previous studies have reported that insulin signaling is modulated by apoE and its genotype [31,32]. Taken together, we suspect that the strong linkage between serum TG levels and roxi/total, [red +oxi]/roxi, and red/roxi may reflect the fluctuations of TG-rich lipoproteins. Moreover, their remnants are affected by pathophysiological redox changes of apoE-Cys-thiol, which is accompanied by the modulation of insulin signaling.

Remnant lipoproteins are highly atherogenic lipoprotein particles [24,33]. Based on this pathophysiological property, we predicted the possibility that the reduction of roxi/total and the increment of [red +oxi]/roxi or red/roxi would exert atherogenic effects or reflect their consequences. Furthermore, we expected that these findings would be observed in patients with atherosclerosis and type 2 diabetes. However, contrary to expectations, our study showed the opposite results, although the TG level in the patients was significantly higher than that in the controls. This discrepancy suggests that pathophysiological redox changes in the serum apoE-Cys-thiol by or for the development of atherosclerosis accompanied by dyslipidemia would be quite different from those by or for the development of dyslipidemia without atherosclerosis.

It is well known that the development of atherosclerosis is caused not only by dyslipidemia but also by chronic inflammation, attributed to oxidative stress [34]. Thus, we predicted that redox-IDX-apoE positively correlates with CRP levels. However, no significant correlation was observed between them, although the CRP levels in the atherosclerotic patients were significantly higher than those in the control subjects. Interestingly, these findings suggest that the promotion of apoE-Cys-thiol oxidation might be independent of the inflammatory response.

Overall, the appropriate usage of these redox ratios of apoE according to the apoE phenotype could allow for the detection of atherosclerosis and its related pathological conditions, such as insulin resistance and dyslipidemia. However, at this stage, it is not certain whether the redox change of serum apoE-Cys-thiol is the cause or effect of these pathological changes. One limitation of the present study is the small sample size; thus, we could not perform a stratified analysis based on the type of dyslipidemia, even though various types of dyslipidemia are induced by type 2 diabetes [35]. Further studies will be necessary to clarify the clinicopathological characteristics of redox changes in serum apoE-Cys-thiol using redox-IDX-apoE.

In conclusion, roxi/total, [red +oxi]/roxi, and red/roxi might be useful indices for estimating the redox status of serum apoE-Cys-thiol.

## Guarantor

K.Y. is the guarantor of the present study.

## Supplementary Material

Supplementary Tables S1-S2

## Data Availability

The data that support the findings of this study are available from the corresponding author, [K.Y.], upon reasonable request.

## Abbreviations

AD: Alzheimer’s disease
apoE: apolipoprotein E
HDL-C: high-density lipoprotein cholesterol
LPL: lipoprotein lipase
TC: total cholesterol
