# Supplementary material for: Redox index of Cys-thiol residues of serum apolipoprotein E and its diagnostic potential
Source: Biosci Rep. 2021 Aug 5;41(8):BSR20211060. doi: 10.1042/BSR20211060 (PMC8350432; doi:10.1042/BSR20211060)
Supplement: Supplementary Tables S1-S2 [file BSR-2021-1060_supp.pdf]

Supplemental Table 1 Relationship between each redox-IDX-apoE candidate and each laboratory test result

Laboratory tests	roxi/total		red/roxi		[red + oxi]/roxi	
	r	*p*	r	*p*	r	*p*
apoAI (mg/dL)	0.040	NS	-0.062	NS	-0.090	NS
apoAII (mg/dL)	-0.107	NS	0.087	NS	0.046	NS
HDL-C (mg/dL)	0.236	<0.001	-0.277	<0.001	-0.256	<0.001
LDL-C (mg/dL)	-0.093	NS	0.044	NS	0.026	NS
non-HDL-C (mg/dL)	-0.275	<0.001	0.247	<0.001	0.237	<0.001
TG (mg/dL)	-0.483	<0.001	0.542	<0.001	0.515	<0.001
TG/HDL-C	-0.384	<0.001	0.447	<0.001	0.425	<0.001
CRP (mg/dL)	0.049	NS	-0.055	NS	-0.054	NS
HbA1c (%)	-0.192	<0.005	0.182	<0.01	0.183	<0.01

NS, not significant.

Supplemental Table 2 Laboratory test results of control subjects and subjects with atherosclerosis

Laboratory tests	control subjects	atherosclerosis subjects
n (% male)	38 (42.1)	16 (50.0)
age (years)	54.7 ± 17.0	56.8 ± 19.6
apoE (mg/dL)	3.05 ± 0.26	2.16 ± 0.15
apoAI (mg/dL)	143.4 ± 3.5	154.7 ± 11.6
apoAII (mg/dL)	32.5 ± 7.5	31.3 ± 9.5
HDL-C (mg/dL)	60.6 ± 14.1	60.3 ± 7.2
LDL-C (mg/dL)	95.0 ± 23.7	104.1 ± 9.6
non-HDL-C (mg/dL)	113.7 ± 26.0	142.4 ± 14.7
TG (mg/dL)	83.7 ± 26.9	170.0 ± 32.8 ***
CRP (mg/dL)	0.083 ± 0.214	1.233 ± 2.997 *
HbA1c (%)	5.04 ± 0.42	5.59 ± 0.62 **

All parameters and ages of subjects are presented as the mean ± SE and mean ± SD, respectively.

*, p<0.05; **, p<0.01; ***, p<0.005.
